# Intergenerational support among migrant families in Europe

**DOI:** 10.1007/s10433-016-0363-6

**Published:** 2016-03-04

**Authors:** Valeria Bordone, Helga A. G. de Valk

**Affiliations:** 1 Centre for Research on Ageing, University of Southampton, Highfield Campus, Southampton, SO17 1BJ UK; 2 Wittgenstein Centre for Demography and Global Human Capital (IIASA, VID/ÖAW, WU), World Population Program, International Institute for Applied Systems Analysis, Laxenburg, Austria; 3 Netherlands Interdisciplinary Demographic Institute (NIDI)/KNAW/UoG, The Hague, The Netherlands; 4 Vrije Universiteit Brussel, Brussels, Belgium

**Keywords:** Ageing, Europe, Immigrants, Intergenerational support, SHARE

## Abstract

Intergenerational support is important throughout the individual life course and a major mechanism of cultural continuity. In this study, we analyse support between older parents and their adult children among international migrant and non-migrant populations in North, Centre and Southern Europe. Data from the Survey of Health, Ageing and Retirement in Europe are used to compare upward and downward practical support, grandparenting, and frequency of contact among 62,213 parent–child dyads. Findings indicate limited differences in support between migrants and non-migrants as well as between migrants of various origins. However, persistent differences in intergenerational support across Europe along a north–south gradient are found irrespective of migrant status.

## Introduction

One of the main demographic changes across Europe is that of population ageing. Novel in this process is that in addition to the increasing number of older people among the majority group, migrant populations in Europe are also ageing. For example, in Germany in 1994, around 6 % of the population aged 60 years or above had a non-German nationality, but this almost doubled in 2004 and rose to 15 % in 2012 (Baykara-Krumme 2008; BPB 2012). Similar patterns are observed in other European countries (Lanzieri 2011; Van Mol and de Valk 2016), and these numbers are expected to rise further in the decades to come (Schimany et al. 2012).

Intergenerational relationships are recognised as a main source of support in later life and they function as a major mechanism of cultural continuity. Research on the majority populations throughout Europe has produced evidence of strong attachment and exchange of support between older parents and their adult children, country differences in the rates and amounts of transfers notwithstanding (e.g. Bordone 2009; Hank 2007; Tomassini et al. 2004). Yet, this has hardly been studied in a comparative framework for migrant families where cohort analyses of differences in the assimilation process prevail. The existing studies on intergenerational support within migrant families have mainly focussed on one reception country or one migrant group (e.g. Attias-Donfut and Wolff 2008; Baykara-Krumme 2008; Cylwik 2002; De Valk and Schans 2008), studied the North-American context (e.g. Becker et al. 2003; Treas and Mazumdar 2004) or referred to families with young(-er) children (e.g. Nauck 2001; Portes and Rumbaut 2006). Furthermore, this literature often covers attitudes towards support or expectations parents have from their children rather than actual support behaviour. Our study complements the existing literature by taking a multiple comparative design in studying older parent–adult child support. Moreover, we consider family intergenerational support in later life in its different dimensions (see Bengtson and Roberts 1991; Roberts et al. 1991 for a typology of family solidarity).

With increasing numbers of older people of migrant origin, it becomes important to know more about the extent to which their support across generations differs from the majority populations. At the same time, it is relevant to go beyond a mere dichotomy between those with and without a migrant background and pay attention to diversity in the regions of origin (Van Mol and de Valk 2016). So far, insights on the extent to which countries of origin shape intergenerational support behaviour among elders who migrated in their lifetime and on how these migrants compare to the majority group in the destination countries have been limited, mainly due to lack of suitable cross-national data. However, migrants in Europe come from a wide variety of origin countries in which prevailing intergenerational support within the family may differ compared to the host country.

The aim of this paper is thus twofold. First, we explore how and to what extent (international) migrant and non-migrant parents in Europe differ in terms of intergenerational support relations with their adult children. Second, we examine the relative importance of region of origin versus region of settlement in the ways intergenerational support takes place in migrant families. Using data from the Survey of Health, Ageing and Retirement in Europe (SHARE), we assess for the first time the relative importance of origin and settlement region, taking into account welfare systems that differently organise support responsibilities between the family and state.

## Background and hypotheses

Family support across generations has developed differently across the globe. Kagitçibasi’s *theory of family change* (1996), presented as a general framework for understanding the systematic variations in the family relationships in different socio-economic and cultural contexts, distinguished between family systems that stress the collective (*relatedness*) and those that focus more on the individual (*separateness*). This theoretical distinction follows in general terms the collectivism-individualism dimension in the index of cultures, as developed by Hofstede (1980, 2001). However, it additionally links the type of family systems to the socio-economic context in which they developed. This second dimension takes into account and goes beyond a mere dichotomy of independence versus interdependence. In countries where state support is absent, families have to rely more on each other to provide the necessary care and (economic) help (Kagitçibasi 1996, 2005). This theoretical framework may help to explain the development of family relationships in migrant families after migration as well as the observed differences in family ties between Northern and Southern Europe. In Nordic countries, welfare systems have taken over part of the care arrangements otherwise shouldered by family members (Dykstra and Fokkema 2011; Esping-Andersen 1990; Reher 1998), moving from a culture of *relatedness* (high levels of intergenerational dependency), to one of *separateness* (where the state is expected to provide material support and family members are supposed to give affective support). Similarly, for migrant families from contexts in which relatedness in family ties prevails (e.g. less developed agricultural societies), the transition to countries with developed welfare systems moves them into a culture of separateness (Kagitçibasi 2005; Kalaycioğlu and Rittersberger-Teliç 2000; Phalet and Schönpflug 2001; Rooyackers et al. 2014).

Theoretically, the effect of migration on support within migrant families has resulted in two contrasting hypotheses (McDonald 2011; Nauck 2007). The first suggests a higher level of cohesion and intergenerational support in migrant families, assuming strong norms and values around intergenerational support. Given that families of migrant origin are often socialised in family systems of relatedness, it is expected that these acquired norms will remain after migration. Families of migrant origin would thus depend more on each other both in the short and longer run. This “place of origin effect” is expected to be similar across Europe. Our first hypothesis, therefore, is that *families of migrant origin will have higher levels of intergenerational support than families of the majority population across Europe (H1.1)*.

The second view suggests that family relations may be more fragile among migrants as a consequence of potential intergenerational and intercultural conflicts (Merz et al. 2009). Migration would thus have a disruptive effect on the support relations between family members, especially between parents and children. The contrasting place of origin effect hypothesis, therefore, is that *families of migrant origin will experience lower levels of support than families of the majority population across Europe (H1.2)*.

Empirical research is still inconclusive about the direction of the migration effect on intergenerational exchange (Baykara-Krumme 2008; De Valk and Schans 2008; Nauck 2007; Nosaka and Chasiotis 2005). Baykara-Krumme (2008) and Schimany et al. (2012) found that intergenerational ties in later life among migrant families in Germany are not too different from those of non-migrant Germans. However, region of origin could affect intergenerational support. Following the theory of family change by Kagitçibasi one would expect that Asia, Africa and South America are more strongly oriented towards the family of relatedness than Europe and that socio-economic necessity of family exchange may prevail there. With migration to Europe and the concomitant decreased economic necessities, families undergo a shift from interdependence to more independent family relations. Studies in the Netherlands suggest that attitudes towards filial obligations are indeed stronger among some migrant origin groups but this is not necessarily related to higher levels of actual support (De Valk and Schans 2008; Schans and de Valk 2012). At the same time, a shift (towards weaker commitments) in values regarding family support was reported among Turkish families (Phalet and Güngör 2009).

Moreover, migrants settling in different regions of Europe encounter different family and welfare support systems. As Kagitçibasi (2005) points out, emotional links between parents and children may continue to prevail in Western urbanised societies. Beyond economic necessity for material support, emotional and associational bonds within the parent–child relationship are based on cultural values and norms (Fuligni et al. 1999). Indeed, several studies have shown the importance of intergenerational support across Europe irrespective of welfare arrangements (e.g. Dykstra et al. 2006; Georgas et al. 2006; Rooyackers et al. 2014; Tomassini et al. 2004). Furthermore striking differences also emerge in the extent to which grandparents engage in care for their grandchildren. In the Mediterranean countries, 40 % of grandparents provide regular grandchild care, compared with 20 % in Nordic countries. However, more North Europeans do active grandparenting compared to their Mediterranean counterparts (Bordone et al. forthcoming; Hank and Buber 2009), possibly reflecting higher maternal employment rates and the more common occasional help to working mothers in those contexts. A north–south gradient also exists in terms of intergenerational contact, with Mediterranean countries reporting higher levels of parent–child contact (e.g. Bordone 2009; Hank 2007).

Theories on immigrant assimilation suggest that migrants adopt the attitudes and behaviour of the majority population over time (Gordon 1964). This has been shown e.g. regarding the labour market, health and mortality (Heath et al. 2008; Rechel et al. 2011). When it comes to core domains of life such as the family, adaptation might be slower and effects of place of residence will only be visible after a longer period (Lesthaeghe 2002). Yet, our migrant sample has been living in the host country for 42 years, on average. We may thus expect that the effect of region of residence does not differ by migration background. Hence, the second hypothesis: *Families of migrant origin (as well as non*-*migrants), living in North*-*western Europe are both less likely to exchange support than is the case for families living in Southern Europe (H2)*.

Families are the source of different types of exchanges between generations (Bengtson 2001; Bengtson and Roberts 1991; Roberts et al. 1991). One may distinguish between *upward support* (i.e. practical help from the child to the parents), *downward support* (i.e. practical and care-related help from the parent to the child), and *associational support* (i.e. frequency of contact). As hinted in the literature reviewed above, both origin and residence effects may differ according to the type of support considered (Attias-Donfut and Wolff 2008). The extent of welfare services may affect how much family members need to rely on each other for practical support (Trommsdorff and Nauck 2005). At the same time, emotional and affectual bonds may remain important. Kagitçibasi (2005) refers to this as the “family model of interdependence”. Thus, place of residence may be more relevant for shaping practical support than for the frequency of contact between parents and children. Moreover, different theories on immigrant adaptation have suggested that adaptation processes are selective, occurring more easily in the practical domains of life (see e.g. Portes and Zhou 1993). Therefore, we hypothesise a selective acculturation effect: *Differences in support between families of migrant origin and the majority group will be more evident for the associational dimension of support than for exchange of practical support (H3)*.

## Data and method

### Sample construction

Data from the Survey of Health, Ageing and Retirement in Europe are used in this study to analyse intergenerational support among migrant and non-migrant families. SHARE is a multidisciplinary dataset, containing information on the country of origin and detailed socio-demographic characteristics of the interviewees and their children (Börsch-Supan et al. 2005; Börsch-Supan and Jürges 2008). For each respondent, we consider the first interview in 2004, 2006 or 2011 wave. We do not use the longitudinal structure of SHARE because migration patterns above the age of 50 are rather limited. We grouped 12 European countries of settlement based on existing welfare and family systems. Denmark and Sweden represent Northern Europe; Austria, Belgium, France, Germany, the Netherlands, and Switzerland represent Central Europe; Greece, Italy, Portugal and Spain represent Southern Europe. Eastern European countries are only considered as countries of origin because for the studied period they were mainly countries of out-migration. This grouping reflects the main differences across Europe in terms of family relations. Moreover, when checked against the individualism-collectivism scale developed by Hofstede (1980), we derive an overall similar grouping.

We retain the sample of parents aged 50+ without missing observations on the dependent variables or on country of birth as this information is central to our analyses. We do not include parent–child dyads living in the same house(hold) given their potentially very different support behaviour. Yet, information is retained on whether at least a child lives with the parent. After these selections, our sample covers a total of 62,213 parent–child dyads. Information about frequency of contact in waves 1 and 2 is available only up to four children (*n* = 59,216). The working sample in our analysis of grandparenting is further reduced by considering only dyads where the child has at least one own child (*n* = 37,244).

As the parent–child dyad is our unit of analysis, more than one child per parent is considered when information is available. All the estimates are therefore obtained adjusting standard errors for correlation among children of the same parent. If migrants had more children than non-migrants or were more likely to live in the same house(hold), there might be a biased representation. Therefore, in additional robustness checks (available on request), we analysed the potential selectivity by migrant origin before selection on geographical proximity. These analyses did not reveal significant differences by migration background (23 % of non-migrant and 25 % of migrant dyads lived in the same household or building). In order to avoid an overrepresentation of parents with more children, we ran the same analyses on a sub-sample where for each parent one child was randomly retained and the results were similar to those reported here.

### Dependent variables

The four-dependent variables in our analyses reflect practical (upward and downward) and associational (i.e. frequency of contact) intergenerational support within the family. Practical support upward the generational lineage is covered in SHARE by asking “Has anyone from outside the household given you [or your husband/wife/partner] help in personal care (e.g. dressing, bathing, etc.); practical household help (e.g. shopping, household chores, etc.); paperwork (e.g. filling out forms, settling financial matters)?”. If the answer is “yes”, it is asked who helped. Up to three persons can be selected and we focussed only on the child in the dyad. Additionally, it is asked which types of help was provided (personal care; practical household help; paperwork) and how often (less than monthly; almost every month; almost every week; almost daily). The answers were recoded into days per year (6; 12; 52; 365) and then summed, resulting in a count variable ranging from 0 to 1095.

Similarly, we constructed a measure of downward support as the number of days that the parent gave practical help in household tasks and/or paperwork to the child in the dyad (outside the household) based on the questions “Who have you helped most often in the last 12 months?”; “Which types of help?; “How often?”. We did not consider personal care as it implies a very particular condition of dependence of the adult child.

The amount of time the parent spent taking care of grandchildren is considered separately as it is one of the main downward transfers in later life. The sample for this analysis is reduced to include only children who have at least one child. SHARE asks the frequency of grandparenting in the previous 12 months (“almost daily; almost every week; almost every month; less often”) for each child. We coded 0 those who have grandchildren, but do not look after them.

The frequency of parent–child contact (either personally, by phone or mail) is measured using seven answer categories on contact frequency in the previous 12 months: “never; less than once a month; about once a month; about once a week; several times a week; every 2 weeks; daily”. Since the questions on practical support and contact refer to both partners, they are attributed to both spouses in cases where they are both interviewed and only one of them answered those questions (this was observed in 160 cases for downward support and 132 cases for contact, which when left out in robustness checks did not change the results).

Table 1 gives an overview of these dimensions by migrant origin.

**Table 1:** Descriptive statistics, by migration background: mean (standard deviation) or percentage and minimum and maximum values of the dependent and independent variables

Variable	Migrant				Non-migrant			
	Mean	S.D.	Min	Max	Mean	S.D.	Min	Max
Dependent variables								
Practical upward support	6.9	68.4	0	1095	5.5	57.7	0	1,095
Practical downward support	1.9	26.6	0	730	2.1	24.3	0	730
Grandparenting^a^	18.4	64.6	0	365	22.5	73.6	0	365
Contact^b^	4.1	1.7	0	6	4.4	1.6	0	6
Parent’s characteristics								
Female (%)	55.5				56.4			
Age	65.7	10.1	50	98	67.0	10.4	50	104
Number of children	3.4	1.9	1	13	3.1	1.6	1	17
Child living with or <5 km (%)	63.7				66.0			
Married (%)	60.5				64.6			
Separated/divorced (%)	17.0				11.5			
Widowed (%)	21.5				22.8			
Never married (%)	1.0				1.1			
Education low (%)	44.0				51.7			
Middle (%)	31.3				29.6			
High (%)	24.7				18.8			
ADL-IADL	0.7	1.7	0	13	0.7	1.8	0	13
Health (1 excellent-5 poor)	3.2	1.1	1	5	3.0	1.1	1	5
Years in the country	41.7	17.9	0	90				
Child’s characteristics								
Daughter (%)	50.7				51.0			
Living with partner (%)	54.8				62.4			
Having own children (%)	56.8				61.4			
Geographical distance: <1 km (%)	10.1				14.7			
1–5 km away (%)	19.4				20.3			
5–25 km away (%)	23.0				26.1			
25–100 km away (%)	16.2				17.5			
100–500 km away (%)	12.8				13.5			
>500 km away (%)	8.6				5.6			
>500 km in another country (%)	10.1				2.3			
N	5439				56,774			

The number of observations refers to the parent–child dyads, considered as unit of analysis. Source: SHARE, authors’ elaboration

^a^ *N*_m_ = 3034; *N*_n−m_ = 34,210

^b^ *N*_m_ = 5093; *N*_n−m_ = 54,123

### Explanatory variables

The main independent variables are the region of residence and, for dyads of migrant origin, the region of origin of the parent. Three regions of residence are distinguished as detailed earlier: Northern, Central and Southern Europe. We consider six origin regions following the classification of countries as suggested by the United Nations Statistics Division (http://unstats.un.org/unsd/methods/m49/m49regin.htm, accessed on September 17, 2015) and based on the size of the sample analysed: North-Western Europe, Southern Europe, Eastern Europe, South America, Africa and Asia. Interviewees with other origin are excluded from the analyses as their samples were numerically too small to be considered as additional geographical regions and too heterogeneous to be grouped with another region in terms of family characteristics or migration patterns. Table 2 summarises the information on regions of residence and of origin in the working sample.

**Table 2:** Descriptive sample by country of origin and country of residence of the parent

Residence	Northern Europe		Central Europe		Southern Europe	
Origin of the interviewed parent	N	%	N	%	N	%
North-West Europe	375	63.3	1586	34.9	51	16.6
Southern Europe	58	9.8	1020	22.5	34	11.0
Eastern Europe	62	10.5	611	13.5	36	11.7
Caribbean-South America	26	4.4	85	1.9	72	23.4
Asia	58	9.8	380	8.4	36	11.7
Africa	13	2.2	857	18.9	79	25.7
Total	592	100	4539	100	308	100
Non-migrants	9176		34,033		13,565	

Classification of the regions of origin based on http://unstats.un.org/unsd/methods/m49/m49regin.htm. The number of observations refers to the parent–child dyads, considered as unit of analysis. Source: SHARE, authors’ elaboration

### Controls

Control variables include socio-demographic characteristics of the child and the parent which were found to be predictors of intergenerational support in previous studies and for which we expect similar effects in migrant and non-migrant families. Descriptives are shown in Table 1 by migration background. Characteristics of the child are: *gender* (1 = son; 2 = daughter), *marital status* (=1 if living with spouse or partner; = 0 otherwise) and *having children* (=1 if has children; = 0 otherwise). For the parent, we control for: *age* (50-65; 66-75; 76 +), *gender* (1 = father; 2 = mother), *marital status* (four dummy variables for married/cohabiting; separated/divorced; widowed; never married), *number of children* (included as continuous variable) and *education* (low if ISCED = 1–2; middle if ISCED = 3–4; high if ISCED = 5–6). All models control for parent–child *geographical distance* in km (<1; 1–5; 5–25; 25–100; 100–500; >500; >500 in another country). A dummy variable indicates whether at least *one sibling lives in the same house(hold)* of the interviewed parent. Given that upward support may be closely linked to a need of the parent deriving from poor health, a variable counting the problems the parent has with *activities of daily living* (ADL) and *instrumental activities of daily living* (IADL) is included. Additionally, we control for *self*-*perceived health* (from 1 = excellent to 5 = poor) of the parent. In case of poor health of the partner, the parent may receive more support from the children. Therefore, we have carried out a robustness check controlling for partner’s health (0 = not married/cohabiting; 1 = having partner in good health; 2 = having partner in poor health) on the sub-sample of married/cohabiting respondents for which there is information about the health status of the spouse (only about 12 %). The results (available on request) are overall the same as those presented.

Pooled models of migrants and non-migrants also include a dummy variable indicating if the parent was *not born in the country of residence*. For migrant parents, we additionally control for the *number of years* they have been living *in the country of residence* (according to the quartile scores on the continuous variable: <31 years; 31–43; 44–56; 57 years or more).

### Method

In order to test our hypotheses, we carry out multivariate analyses on the pooled sample and on the two sub-populations defined by migration background separately. Zero-inflated negative binomial models (*zinb* in STATA) are used to study practical support and grandparenting, since the majority of the sample has value 0 in the outcome variable (i.e. no support). Theory suggests that the excess zeros are generated by a separate process from the count values and that they can be modelled independently. Zinb models predict a first part where all the variables of main interest as well as controls are included to estimate the association between the dependent variable and each independent variable considered (a negative binomial model to model the count process); a second part (a binary model, available from authors upon request) predicts the 0, thus it tells which of the variables considered are more likely to predict a 0 outcome. For example, the coefficient of parent’s health on upward support would indicate that a person in poor health is less likely to have a “0” (i.e. a condition of poor health increases the probability of receiving support). The expected count is expressed as a combination of the two processes. Alternative methods to the zinb could be OLS—however, count data are highly non-normal; Zero-inflated Poisson—it would be better if data were not overdispersed; Ordinary Count Models—more appropriate if there would not be excess zeros. The Vuong test, comparing the zinb with ordinary negative binomial regression models, has significant *z* test (<0.000) in all the models, indicating that the zero-inflated model is preferred.

Due to the nature of the variable measuring parent–child contact, an ordinal logistic model is used to analyse contact frequency.

## Results

Descriptively, the frequency of upward and downward practical support does not significantly differ between non-migrant and migrant families. Grandparenting and face-to-face or telephone contact occur significantly more often among the majority population than is the case for parent–child dyads of migrant origin (results available on request).

In order to test Hypothesis H1, we carry out regression models on the four-dependent variables for the pooled sample, controlling for migration background (Table 3a). Parent–child dyads where the parent migrated (out of the country of birth) report significantly higher levels of downward practical support and time for grandparenting and also have more frequent contact, supporting Hypothesis H1.1. These findings are opposite to the patterns found in the descriptive analyses suggesting that compositional differences between migrant and non-migrants are important. The positive association between migrant origin and upward support, significant only at 10 %, is in line with the descriptive results.

**Table 3:** Multivariate results on (a) the pooled sample of migrants and non-migrants and (b) on the migrant and non-migrant samples separately

(a)	Practical upward support	Practical downward support	Grandparenting	Contact
Migrant (Ref.: not)	1.18^+^	1.57***	1.18***	1.16***
Residence (Ref.: Northern Europe)				
Central Europe	1.63***	1.33***	1.82***	0.75***
Southern Europe	1.90***	1.98***	4.08***	2.36***
Female (male)	0.87*	1.18**	1.21***	1.35***
Age 66–75 (Ref.: 50–65)	0.98	1.19*	1.03	0.82***
75^+^	1.12	1.51***	0.94	0.78***
Number of children (Ref.: 0)	0.96*	0.82***	0.86***	0.79***
Children living with or <5 km	0.99	1.44***	1.24***	1.11***
Divorced (Ref.: married)	1.06	1.15	0.90*	0.51***
Widowed	1.46***	1.31**	0.91*	0.86***
Never married	3.50***	2.15**	0.71*	0.46***
Education (Ref.: low) middle	0.62***	0.89^+^	0.92**	1.02
High	0.58***	0.89	0.89***	1.12***
ADL-IADL	1.16***	1.10**	1.04**	0.96***
Self-perceived health	1.13***	1.08*	1.02	0.97***
Daughter (son)	1.70***	1.24***	1.30***	1.61***
Child living with partner (not)	0.9	0.91	0.82***	1.08***
Child has own children (not)	0.77***	1.07	(omitted)	1.03
Geographical distance	0.69***	0.81***	0.70***	0.66***
Constant	94.49***	41.63***	102.78***	
cut1_cons				0.00**
cut2_cons				0.01***
cut3_cons				0.02***
cut4_cons				0.03***
cut5_cons				0.10***
cut6_cons				0.58***
N	62,213	62,213	37,244	59,216
Ll	−26759.17	−22455.77	−78880.25	−90358.66
Vuong	20.02***	23.07***	56.21***	

(b)	Practical upward support		Practical downward support		Grandparenting		Contact	
	Migrant	Non-migrant	Migrant	Non-migrant	Migrant	Non-migrant	Migrant	Non-migrant
Residence (Ref.: Northern Europe)								
Central Europe	1.77*	1.59***	0.92	1.33***	1.65***	1.82***	0.68***	0.75***
Southern Europe	1.92	1.96***	0.43	2.03***	2.79***	4.13***	1.22	2.43***
Origin (Ref.: North-West Europe)								
Southern Europe	2.54***		1.02		1.24^+^		1.42***	
Eastern Europe	1.83*		0.39^+^		1.55**		0.99	
South America	0.93		0.03***		1.82*		1.2	
Asia	0.55		0.3^+^		1.64*		1.28*	
Africa	6.29***		0.82		1.41*		1.37***	
Female (male)	0.69^+^	0.88*	0.88	1.20**	1.07	1.22***	1.58***	1.34***
Age 66–75 (Ref.: 50-65)	1.2	1.04	0.39^+^	1.27**	1.21^+^	1.02	0.91	0.82***
75^+^	1.96*	1.18*	0.66	1.61***	1.09	0.94	0.94	0.79***
Number of children (Ref.: 0)	0.95	0.95*	0.71*	0.81***	0.88***	0.86***	0.82***	0.78***
Children living with or <5 km	0.61*	1.03	1.94	1.39***	0.86	1.29***	1.06	1.11***
Divorced (Ref.: married)	0.89	1.08	1.7	1.05	1.12	0.87**	0.66***	0.49***
Widowed	1.28	1.49***	0.81	1.26*	1.12	0.91**	0.82**	0.86***
Never married	0.51	3.84***	0.06	2.26**	0.67	0.71*	0.50**	0.46***
Education (Ref.: low) middle	0.37***	0.65***	0.44^+^	0.86*	0.94	0.93*	1.03	1.03
High	0.57*	0.60***	0.99	0.89	0.64***	0.92*	1.15*	1.14***
ADL-IADL	1.13**	1.16***	0.87	1.11**	1.11^+^	1.04*	0.97^+^	0.96***
Self-perceived health	1.21*	1.13***	1.74*	1.05	0.98	1.01	1.01	0.96***
Years in the country 30-42 (Ref.: <30)	0.49*		0.20**		0.94		0.91	
43–56	0.36**		0.24**		0.86		0.75***	
57^+^	0.51*		0.22**		1.17		0.64***	
Daughter (son)	1.38^+^	1.70***	1.05	1.26***	1.48***	1.28***	1.42***	1.63***
Child living with partner (not)	1.51*	0.87*	1.25	0.88^+^	0.75**	0.82***	1.08	1.08***
Child has own children (not)	0.54**	0.78***	2.12^+^	1.05	(omitted)	(omitted)	1.12^+^	1.02
Geographical distance	0.62***	0.70***	1.01	0.80***	0.61***	0.71***	0.67***	0.65***
Constant	359.32***	82.81***	64.54***	52.65***	265.82***	95.82***		
cut1_cons							0.01***	0.00***
cut2_cons							0.01***	0.01***
cut3_cons							0.02***	0.02***
cut4_cons							0.04***	0.03***
cut5_cons							0.13***	0.10***
cut6_cons							0.63*	0.56***
N	5,439	56,774	5,439	56,774	3,034	34,210	5,093	54,123
ll	−2363.16	−24344.03	−1479.26	−20924.37	−5984.14	−72850.84	−8350.94	−81913.51
Vuong test z	7.17***	19.08***	5.08***	22.32***	16.22***	53.92***		

Odds ratios. Source: SHARE, authors’ elaboration

*** *p* < 0.001; ** *p* < 0.01; * *p* < 0.05; ^+^ *p* < 0.1

Table 3b shows the results of the models on dyads of migrant and non-migrant origin separately to further explore H1 by accounting for the region of origin, and to test H2 and H3. We find that migrant dyads with Southern and Eastern European origin as well as those with African origin tend to report higher upward support than is the case for those with North-western European origin. Although Asian origin seems to be related to lower upward support, the sample size is small (only about 5 % of migrants with Asian origin in the sample receive practical support from the child). Downward support is reported to be consistently lower for all origins compared to those whose parents have a North-western or Southern European origin; however, differences between migrants of various origins are limited. Grandparenting is lowest among migrants of North-western European origin but those of Southern European origin do not statistically differ from this (comparison) group. Finally, frequency of contact is higher among migrants of Mediterranean, Asian and African origin than is the case for North-western Europeans. The small sub-samples of dyads exchanging support among migrants by place of origin require a careful interpretation of these results, especially when not statistically significant as they may reflect a low statistical power.

Marked differences emerge between the regions of residence in all the dimensions of intergenerational support considered in the pooled models, in line with Hypothesis H2. Both upward and downward support as well as grandparenting are significantly more frequent the more southern the region is. We also find more contact between generations in the Mediterranean than in the rest of Europe. However, in this case Central Europe is not showing an in-between position as it has, on average, lower contact than the northern region. The findings for the non-migrant and migrant samples (Table 3b) confirm such a north–south gradient, with a few exceptions of non-significant effects among the parent–child dyads with migration background in the south most likely due to the smaller sample size. Similarly, it is difficult to interpret the non-significant effect of place of residence found for the migrant sample for practical downward support as it is likely to derive from a low statistical power.

We do partly confirm Hypothesis H3 regarding selective acculturation. We do not find a significant difference in practical upward support between migrant and non-migrant populations. However, the effect of having a migration background is strong in distinguishing not only parent–child dyads’ frequency of contact, but also downward practical support and grandparenting. We acknowledge that grandparenting may represent both practical help and emotional-associational bonds.

All control variables show effects in line with what we know from the literature. We note that the associations found in the literature and in this study for the non-migrant population also largely hold for the population of migrant origin. Interestingly, the longer the parent has been in the host country, the lower intergenerational exchange is, but the effect is not always linear.

## Discussion

Intergenerational support is central in both academic and public debates on the role of the family in times of increased longevity, and it is crucial for those involved as well as for society at large. Although studies have advanced our understanding of relationships between older parents and adult children of the majority groups across Europe, much less is known on those of migrant origin in a comparative perspective. A more nuanced understanding of such intergenerational support is essential to capture the increasing diversity of the European population. Our study considered a double comparative perspective. First, we compared migrant and non-migrant parent–child dyads. Second, we focussed on how the regions of settlement and origin may affect parent–child dyads with a migration background. In this respect, Europe is a natural experimental setting where different welfare state provisions and norms regarding the family prevail. Using cross-country comparable data from SHARE, we were able to explore upward and downward practical support, grandparenting and contact between parents with and without an international migration background and their adult children across Europe.

Our findings showed that overall more support is exchanged in migrant families than in the majority population across Europe, suggesting strong intergenerational bonds and/or needs in migrant families. At the same time, however, similarities rather than differences emerged in the socio-demographic determinants of parent–child support by migration background. In particular, the place of residence plays a significant and similar role in explaining the amount of support exchanged between family members for the majority population and families of migrant origin. This result suggests the importance of the context of settlement for support exchange. Our analyses implicitly took policies into account by following the often suggested diversity in welfare systems and family norms across regions of residence in Europe, but we could not distinguish between the two effects.

Differences between migrants of various origins were found to be limited, suggesting a more important effect of country of settlement than origin on intergenerational support. This could partially result from the fact that most children in our study were born in the country of residence and the studied migrant families resided a long period in the country. Since intergenerational relations here considered are dyadic, the effect we find for country of residence may actually account for the child’s embeddedness in the culture and welfare system of the country of residence. Yet, origin does have an effect and, in this respect, our results are in line with earlier studies (e.g. de Valk and Saad 2008, reporting that intergenerational support in South America changes direction over the life course and children are expected to take care of parents later in life). In this sense, cultural norms on parent–child support seem to continue after migration, irrespective of region of residence or welfare system.

The greater use of grandparental childcare among certain origins may also point to the role of family norms on raising children (e.g. Kagitçibasi 2005; Treas and Mazumdar 2004). At the same time, it may also point to higher economic necessity among these families calling for more comparative work.

We interpret the differences in the type of support exchanged between parents and children as the more practical dimensions in the relationship are more likely to adapt to the context of residence, while the associational bonds remain according to values and norms, as was suggested in the literature review. Yet, we acknowledge that we lack information on support before and after migration and information about support behaviours in the country of origin. Longitudinal data measuring norms, values and support behaviour before and after migration would be helpful to study whether migrants are more likely to move to countries with similar family systems.

The data used in our study have some limitations. First, the migrant sample is relatively small and we had to group migrant origins by rather broad regional categories. Although this grouping is justified in terms of more general theories on family relations worldwide, it may not capture full cross-country diversity. Second, our analyses pointed to the existence of transnational family relations, but we could not fully capture it: in about 10 % of our migrant sample parent–child dyads lived in different countries. Transnational families are an increasingly important group (Mazzucato et al. 2015), and the way in which they negotiate support across borders as well as the interplay between policies and personal ties needs more attention. Studying transnational relations may also shed partial light on the potentially selective return migration of older people, their care needs, and support norms.

Third, our sample of migrant families was composed mainly of Europeans, who were often higher educated. This potentially points to a selection of interviewees with a migration background in SHARE, likely to be well integrated in the settling environment (e.g. interviews are only carried out in the country of residence language). Yet, the current reality of European mobility deserves more attention than it has received so far, with existing work on intergenerational support in migrant families mainly focussing on non-Europeans. Moreover, ageing migrants in Europe are on average long-term migrants who are possibly integrated in the society where they moved to. At the same time, those who decided to stay in Europe may potentially have different support norms than those who returned to their countries of origin and as such are not part of our analyses. This calls for better data on migrants who are followed over the life course in order to also capture return migration. Finally, from a methodological point of view, it would have been interesting to consider interaction effects of origin and destination regions, unfortunately impossible due to the limited sample size per region.

Our findings and the limitations of this study suggest the need for extending these analyses. First, accounting for norms (e.g. via an explicit inclusion of family norms in the analyses) could point to the extent that parent and child are more oriented towards the collective or individual and indicate their willingness to provide support to each other. Second, including policy indicators on formal care availability to both children and older family members in the countries under study could increase our understanding of macro-level drivers of the dyadic forms of support (e.g. Bordone et al. forthcoming; Brandt and Deindl 2013). It is therefore advocated to collect more country data that allow cross-country comparisons in order to pay full justice to individual country differences. The worldwide population ageing process puts into question the role and function of intergenerational ties in later life across different ethnic origin. Despite the fact that Europe is one of the main destination areas for migrants in the world, little is still known on intergenerational support between older parents and adult children in families with a migration background. The diversity in regions of origin as well as of destination in SHARE allowed us to explore these associations. Unravelling macro-level effects, how different types of support interact, and the effect that the economic crisis in Europe may have on parents and children of migrant (and non-migrant) origin remain important subjects for further study.
